# Investigation of the preventive action of rebamipide versus cadmium nephrotoxicity effect in rats

**DOI:** 10.1007/s11845-025-04204-y

**Published:** 2025-12-16

**Authors:** Amr A. Abd Ellah, Reem M. Hazem, Amany M. Gad, Yasser M. Moustafa

**Affiliations:** 1 Pharmacology and Toxicology Department, Faculty of Pharmacy, Suez Canal University, Ismailia, Egypt; 2 Pharmacology and Toxicology Department, Egyptian Drug Authority, Cairo, Egypt; 3 Pharmacology and Toxicology Department, Faculty of Pharmacy, Kantara Department, Sinai University, Ismailia, 41636 Egypt; 4 Pharmacology and Toxicology Department, Faculty of Pharmacy, Badr University in Cairo, Cairo, Egypt

**Keywords:** Cadmium chloride, Inflammation, Nephrotoxicity, Oxidative stress, Rebamipide

## Abstract

**Background:**

Cadmium (Cd) is a major environmental and industrial pollutant that exhibits a significant health risk to humans and animals. Renal toxicity is a prevalent adverse effects of cadmium exposure. Rebamipide (REBA) is pharmacological agent known for its potent antioxidants properties.

**Aim:**

This study aimed to investigate the potential reno-protective effects of rebamipide against cadmium-induced nephrotoxicity.

**Main methods:**

Fifty rats were allocated into five groups; (1) a normal control group; (2) cadmium chloride group (5 mg/kg, single dose, i.p.); (3) a group treated with rebamipide (100 mg/kg, p.o.) for nine days (seven days before and two days after a single cadmium dose); (4) a group treated with rebamipide (200 mg/kg, p.o.) for nine days (seven days before and two days after a single cadmium dose); and (5) a group treated with rebamipide only (200 mg/kg, p.o.) for nine days.

**Key findings:**

Cadmium administration significantly increased serum urea, creatinine, and the renal injury marker KIM-1. It also elevated inflammatory mediators, including tumor necrosis factor-α (TNF-α), nuclear factor kappa B (NF-κB), and inducible nitric oxide synthase (iNOS). Concurrently, cadmium halted catalase (CAT), superoxide dismutase (SOD) activities, and reduced glutathione (GSH) level, while increasing malondialdehyde (MDA) content. Furthermore, cadmium dysregulated autophagy, decreasing Beclin-1 and increasing microtubule-associated protein 1 light chain 3 (MAP-LC3) levels. Treatment with rebamipide effectively improved renal histology, reduced inflammatory markers, and restored the balance of all the aforementioned oxidative and autophagic parameters.

**Conclusion:**

Rebamipide acts as a promising preventive agent against cadmium-induced nephrotoxicity, through its potent antioxidant and anti-inflammatory mechanisms.

**Graphical abstract:**

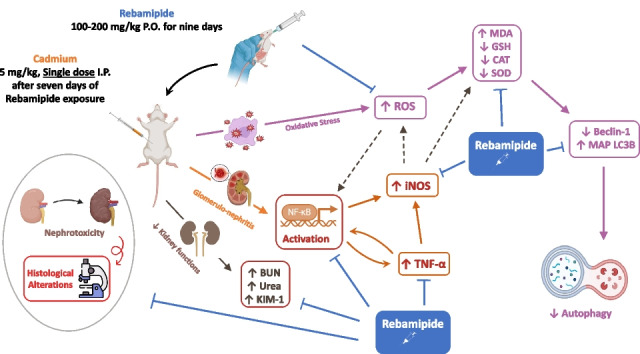

## Introduction

Heavy metals are generally defined as elements with a high density at least five times greater than that of water [1]. Their widespread use in industry and all fields represents a danger to human health [2]. Whether released from industrial or natural sources, heavy metals can seriously endanger human life in a manner similar to other hazardous compounds [3]. Heavy metals are among the most prevalent environmental contaminants [4]. Cadmium (Cd) is a particularly hazardous heavy metal that poses a serious danger to both the environment and human health [5]. Unlike many other heavy metals, cadmium exerts toxic effects at relatively low concentrations [6]. According to the Agency for Toxic Substances and Disease Registry (ATSDR, 2022), cadmium is ranked as the seventh most hazardous substance, reflecting its significant risk and according to the International Programme on Chemical Safety (IPCS), cadmium is recognized as a mutagen and human carcinogen [7]. The major pathway of human exposure to cadmium is through gastrointestinal absorption [8]. Numerous organs, including the kidneys, liver, bones, cardiovascular system, and endocrine system, are affected negatively by cadmium toxic effects [9]. The molecular mechanisms of cadmium-induced nephrotoxicity are complex. When Cd is absorbed into the bloodstream, the liver quickly absorbs it and binds it to metallothionein (MT). The cadmium-metallothionein complex (Cd-MT) is released into the bloodstream and is efficiently filtered by the glomeruli due to its low molecular weight [10]. The proximal tubule reabsorbs the Cd-MT complex leading to the intracellular accumulation of cadmium [10].The kidneys produce metallothionein as a protective mechanism, but there is a certain limit; when the cadmium concentration rises, tubular damage begins to occur [10]. Additionally, Autoantibodies against metallothionein (MT) may be produced, which can interfere with cadmium detoxification and exert tubulotoxic effects [11]. The S1 and S2 segments of the proximal tubule are particularly vulnerable to cadmium accumulation, which leads to intracellular damage and tubular injury [12]. This injury results in a reduced glomerular filtration rate (GFR), which directly elevates serum urea and creatinine concentrations [13]. Also, Cadmium accumulation in the kidney leads to the formation of free radicals, resulting in increased apoptosis, inflammation, lipid peroxidation, membrane protein damage, and glomerular dysfunction [14]. Free radicals cause activation of the nuclear factor kappa B (NF-κB) signaling pathway, which leads to an increase in tumor necrosis factor-alpha (TNF-α) and inducible nitric oxide synthase (iNOS) [15].

Rebamipide (REBA) (is a gastro-protective drug used for the treatment of gastric ulcers [16]. REBA received approval in Japan in September 1990 and has been available since December 1990 [17]. REBA works by increasing prostaglandin and blocking free radicals [18]. REBA is also classified as an anti-inflammatory agent due to its ability to scavenge free radicals, superoxide anions, and hydroxyl radicals [19, 20]. It aids in the inhibition of tumor necrosis factor-alpha (TNF-α) release [21]. REBA prevents the harmful effect of macrophage by inhibiting nuclear factor kappa B (NF-κB) activation [22]. The higher the dose of rebamipide, the greater its inhibitory effect on nuclear factor kappa B (NF-κB) formation [23]. REBA has anti-inflammatory, antioxidant and anti-apoptotic effects that mediate its renoprotective effects [24]. It was reported that, its antioxidant effect involves the restoration of Cu–Zn superoxide dismutase and glutathione S-transferase enzymes [25].

The main objective of this study is to evaluate the protective effects of Rebamipide against cadmium-induced nephrotoxicity in rats and to determine the underlying mechanisms involved. Although rebamipid gastroprotective properties are well-documented, its efficacy in attenuating heavy metal-induced renal damage has not been thoroughly investigated. This study investigated the precise renoprotective mechanisms of rebamipide against cadmium-induced toxicity, focusing specifically on the dose-dependent modulation of oxidative stress, inflammatory mediators (NF-κB/TNF-α/iNOS), and autophagy pathways, thereby revealing novel aspects of its pharmacological activity.

## Materials and methods

### Animals

Fifty male albino Wistar rats, weighing 210–280 g, were obtained from the Egyptian Society for Biological Products (Vacsera, Egypt). Rats were randomly allocated into five equal groups of ten rats each. The animals were maintained under controlled environmental conditions: ambient temperature at 24 ± 1 °C, relative humidity at 50 ± 5%, and a standardized 12-h light/dark cycle (lights on from 07:00 to 19:00). All experiments were conducted in accordance with the ARRIVE guidelines and the NIH Guide for the Care and Use of Laboratory Animals (8th edition, 2011). The study protocol was approved by the Research Ethics Committee of the Faculty of Pharmacy, Suez Canal University (Approval No. 202207MA2).

### Experimental layout

The aim of this study was to evaluate the protective effects of different doses of rebamipide against cadmium-induced nephrotoxicity. Fifty rats were divided into five experimental groups. The normal group was the first Control group. The next group, designated as the cadmium group (disease model), received a single intraperitoneal dose of cadmium chloride (CdCl₂) at 5 mg/kg along with daily saline injections [26]. The third group, which served as the low-dose treatment group, received rebamipide (100 mg/kg, p.o.) [27] for 9 days, starting 1 week before and continuing for 2 days after single intraperitoneal dose of CdCl_2_ (5 mg/kg). The fourth group received the high dose of rebamipide (200 mg/kg, p.o.) for 9 days, starting 1 week before and continuing for 2 days after a single intraperitoneal dose of CdCl₂ (5 mg/kg). The last group received the high dose of rebamipide only (200 mg/kg, p.o.) for nine days.

### Induction of toxicity

Nephrotoxicity was induced by a single intraperitoneal dose of cadmium chloride 5 mg/kg [26].

### Serum and renal homogenate preparation

At the end of the experiment, blood samples were collected from the rats under light anesthesia induced by an intraperitoneal injection of a mixture of ketamine (75 mg/kg) and xylazine (10 mg/kg). Blood was allowed to clot for 30 min. Blood samples were then centrifuged for 15 min to separate the serum, which was subsequently used for biochemical analysis. Serum creatinine and urea are the markers of kidney functions. The animals were then euthanized by decapitation. The kidneys were separated, weighed and homogenized in Phosphate buffered saline to obtain a 10% homogenate, which was stored frozen at—80 °C for subsequent biochemical analyses.

### Renal histopathological examination

Each kidney was fixed in 10% formalin and embedded in paraffin. Sections were cut at 3–4 µm thickness. Each part was stained with Hematoxylin and Eosin (H&E) and examined under a light microscope at various magnifications (e.g., 100x, 400x) for histological examination. Histological examination revealed tubular epithelial abnormalities, including flattening of the epithelium, loss of brush border, and cellular necrosis. A blinded pathologist, unaware of the treatment groups, evaluated the sections. A semi-quantitative scoring system ranging from 0 to 4 was used, where 0 indicated no damage and 4 represented severe damage. The histopathological changes and renal damage were thoroughly examined and assessed against the treatment effects under light microscopy at 16 × and 40 × magnification.

### Biochemical measurements

#### Detection of serum biomarkers

Serum urea and creatinine levels were analyzed using Biodiagnostic kits (Cairo, Egypt) with catalog numbers UR2110 and CR1250. The results were expressed in mg/dL for serum creatinine and blood urea nitrogen.

### Detection of anti-oxidant biomarkers

Rat reduced glutathione (GSH), superoxide dismutase (SOD), and catalase levels were analyzed using MyBioSource ELISA kits (San Diego, CA, USA) with catalog numbers MBS724319, MBS036924, and MBS006969, respectively, and the results for reduced glutathione (GSH) were expressed in µg/g tissue. Superoxide dismutase (SOD) and catalase (CAT) activities were expressed in U/g tissue. Malondialdehyde (MDA) was assessed using Lifespan bioscience** (**LSBio Sciences, WA, US) catalog number:LS-F28018. The results were expressed in ng/g tissue.

#### Detection of inflammatory biomarkers

The involvement of inflammation in cadmium-induced renal toxicity was assessed by measuring tumor necrosis factor-alpha (TNF-α), inducible nitric oxide synthase (iNOS), and nuclear factor kappa B (NF-κB). All these markers were analyzed using MyBioSource kits (San Diego, CA, USA) with catalogue numbers MBS355371, MBS723326, and MBS453975 for each marker, respectively. The outcomes were expressed in ng/mg tissue for iNOS and NF-κB, and in pg/g tissue for TNF-α. Renal kidney injury molecule-1 (KIM-1), as an inflammatory marker, was assessed using an ELISA kit from EIAab (Wuhan, Hubei, China), catalog number E0785r. The results for KIM-1 were expressed in ng/g tissue.

#### Detection of autophagy biomarkers

Beclin-1 and microtubule-associated proteins 1A/1B light chain 3 (LC3) were evaluated using MyBioSource ELISA kits (MyBioSource, California, San Diego, USA) with catalogue numbers MBS733192 and MBS938189, respectively. The results were expressed in pg/g tissue for Beclin-1 and ng/g tissue for MAP1LC3B.

### Statistical analysis

Results were presented with mean and standard deviation using GraphPad Prism software (version 9.0.0 for Windows). Comparisons between multiple groups were conducted by one-way analysis of variance (ANOVA) followed by Tukey–Kramer’s post-hoc test for pairwise comparisons. A probability value (p-value) of less than 0.05 was considered statistically significant.

## Results

### Effect of rebamipide on cadmium-induced nephrotoxicity through assessment of kidney injury molecule-1 (KIM-1), serum urea, and creatinine levels

Administration of cadmium (5 mg/kg) caused a considerable elevation in KIM-1, creatinine, and urea by 384.3%, 61.7%, and 96.2%, respectively, compared to the normal control (*p* < 0.05). Concurrent administration of rebamipide (100 and 200 mg/kg/day, p.o., for 9 days) dose-dependently attenuated these elevations. The low dose (100 mg/kg) reduced KIM-1, creatinine, and urea levels by 39.6%, 44.5%, and 49.9%, respectively. The high dose (200 mg/kg) produced more pronounced reductions in KIM-1, creatinine, and urea by 57.9%, 41%, and 31.3%, respectively (Table 1). These findings demonstrate the protective effect of rebamipide against cadmium-induced renal functional impairment.

**Table 1:** The renoprotective action of rebamipide was evaluated against cadmium-induced renal toxicity. Treatments involved oral administration of two doses: rebamipide at 100 mg/kg/day and 200 mg/kg/day. The dosing regimen spanned nine days in total, commencing one week prior to a single intraperitoneal injection of cadmium (5 mg/kg) and continuing for two days thereafter.The protective effects were assessed by measuring the levels of kidney injury molecule-1 (KIM-1), serum creatinine, and urea

Parameter	Kim 1 (Ng/g tissue)	Creatinine (mg/dl)	Urea (mg/dl)
Treatment			
CONT-saline	6.88 ± 0.3430	0.5367 ± 0.03882	51.83 ± 5.913
Cd (5 mg/kg)	33.32 ± 3.172^a^	0.8650 ± 0.1223^a^	101.7 ± 14.56^a^
REBA 100 + cd	20.13 ± 2.089^ab^	0.4800 ± 0.06066^b^	51.00 ± 2.898^b^
REBA 200 + cd	14.02 ± 1.361^abc^	0.5100 ± 0.03578^b^	69.83 ± 6.969^abc^

Each value represents the mean and standard deviation of the mean of 10 rats

Different letters (a, b, c) denote statistically significant differences (*p* < 0.05) among the treatment groups as determined by one-way ANOVA followed by the Tukey–Kramer test

### Effect of rebamipide on cadmium-induced nephrotoxicity: focus on the renal oxidative stress markers

**Fig. 1:**
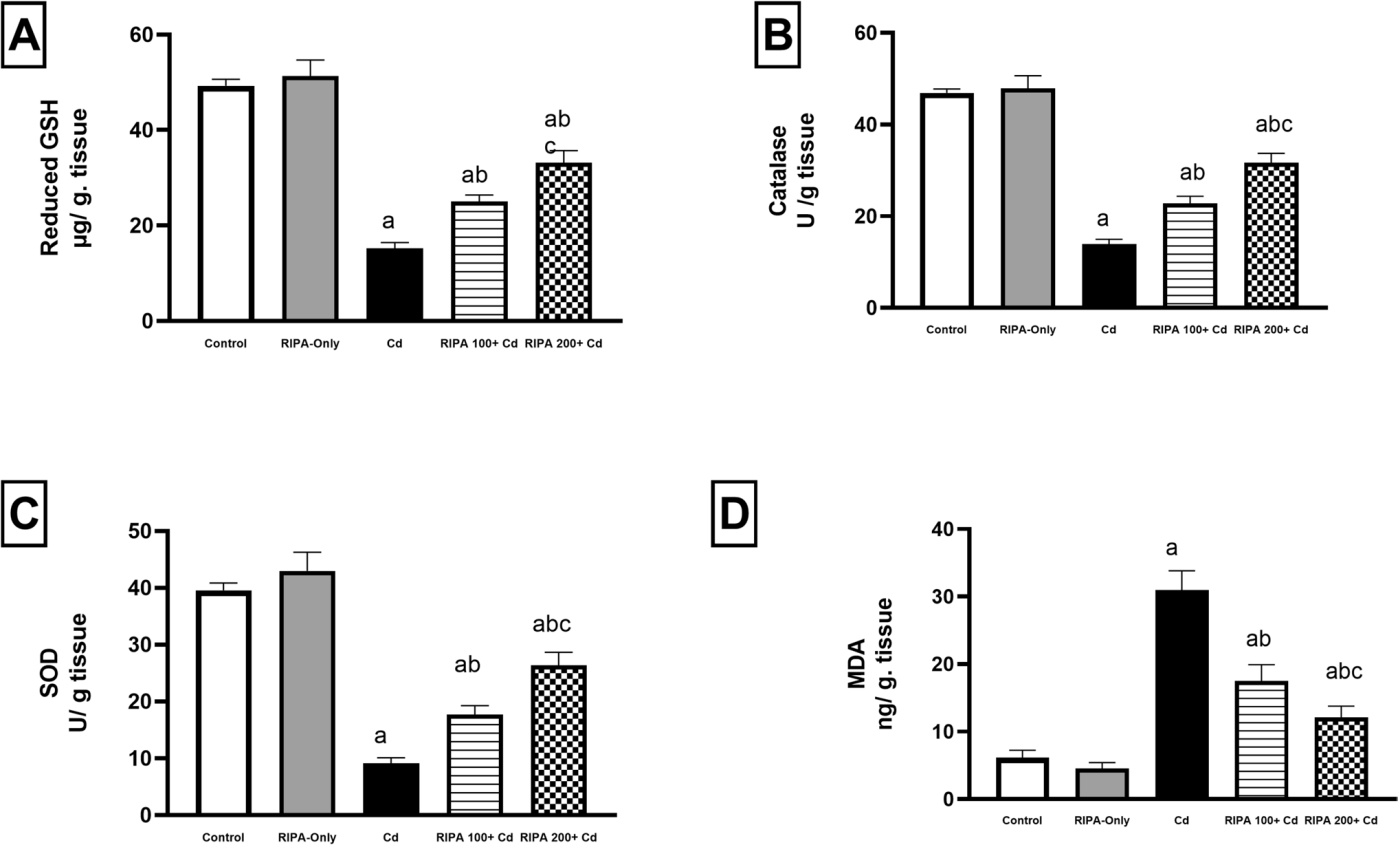
(**A**-**D**) Renoprotective effect of rebamipide (100 and 200 mg/kg/day for 9 days: 7 days before and 2 days after a single intraperitoneal dose of Cadmium chloride [5 mg/kg]) on renal (**A**) glutathione, (**B**) catalase, (**C**) Superoxide dismutase, and (**D**) malondialdehyde levels in cadmium-induced renal toxicity. Values represent the mean ± SD (*n* = 10). Different superscript letters (a, b, c) indicate significant differences among groups (*p* < 0.05, one-way ANOVA followed by Tukey–Kramer post-hoc test)

Administration of cadmium (5 mg/kg) increased reactive oxygen species (ROS) production. This result caused a considerable depletion of reduced glutathione (GSH), catalase (CAT), and superoxide dismutase (SOD), along with elevated malondialdehyde (MDA) levels This led to reductions in GSH, CAT, and SOD by 69.2%, 70.3%, and 76.9%, respectively, and a significant increase in MDA by 403.6% compared to the control group (*p* < 0.05). Concurrent administration of rebamipide at the low dose (100 mg/kg) increased GSH, CAT, and SOD by 65.2%, 64%, and 93.8%, respectively, while decreasing MDA by 43.5% compared to the cadmium-only group. The high dose (200 mg/kg) produced more pronounced increases in GSH, CAT, and SOD by 119.1%, 127.2%, and 188.5%, respectively, and a significant decrease in MDA by 61% Fig. 1. These results demonstrate that rebamipide effectively counteracted cadmium-induced oxidative stress in renal tissue.

### Effect of rebamipide on renal inflammatory markers

**Fig. 2:**
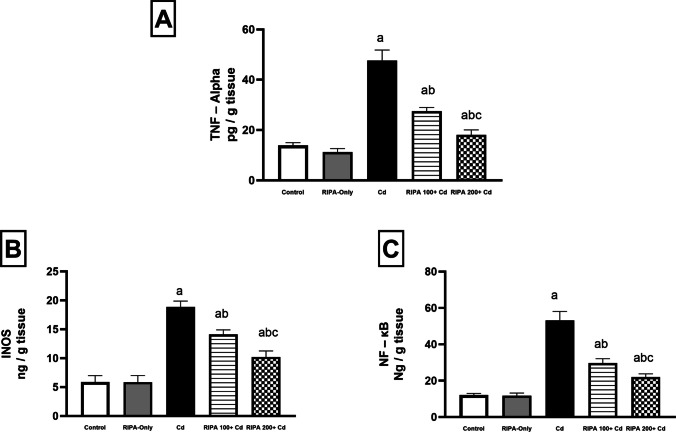
(**A**-**C**) Renoprotective effect of rebamipide (100 and 200 mg/kg/day for 9 days: 7 days before and 2 days after a single intraperitoneal dose of Cadmium chloride [5 mg/kg]) on renal (**A**) tumor necrosis factor-α (TNF-α), (**B**) inducible nitric oxide synthase (iNOS), and (**C**) nuclear factor kappa B (NF-κB) in cadmium-induced renal toxicity. Values represent the mean ± SD (*n* = 10). Different superscript letters (a, b, c) indicate significant differences among groups (*p* < 0.05, one-way ANOVA followed by Tukey–Kramer post-hoc test)

Administration of cadmium (5 mg/kg) increased renal inflammatory mediators, including tumor necrosis factor-alpha (TNF-α), inducible nitric oxide synthase (iNOS), and nuclear factor kappa B (NF-κB) levels by 241.8%, 220%, and 337.3%, respectively (*p* < 0.05). Concurrent administration of rebamipide at the low dose (100 mg/kg) decreased TNF-α, iNOS, and NF-κB levels by 42.4%, 25.2%, and 44%, respectively, compared to the cadmium-only group. The high dose (200 mg/kg) produced more pronounced reductions in TNF-α, iNOS, and NF-κB by 62%, 45.9%, and 58.6%, respectively Fig. 2. This suggests that rebamipide exerts anti-inflammatory effects by modulating the NF-κB pathway.

### Effect of rebamipide on renal autophagy markers

**Fig. 3:**
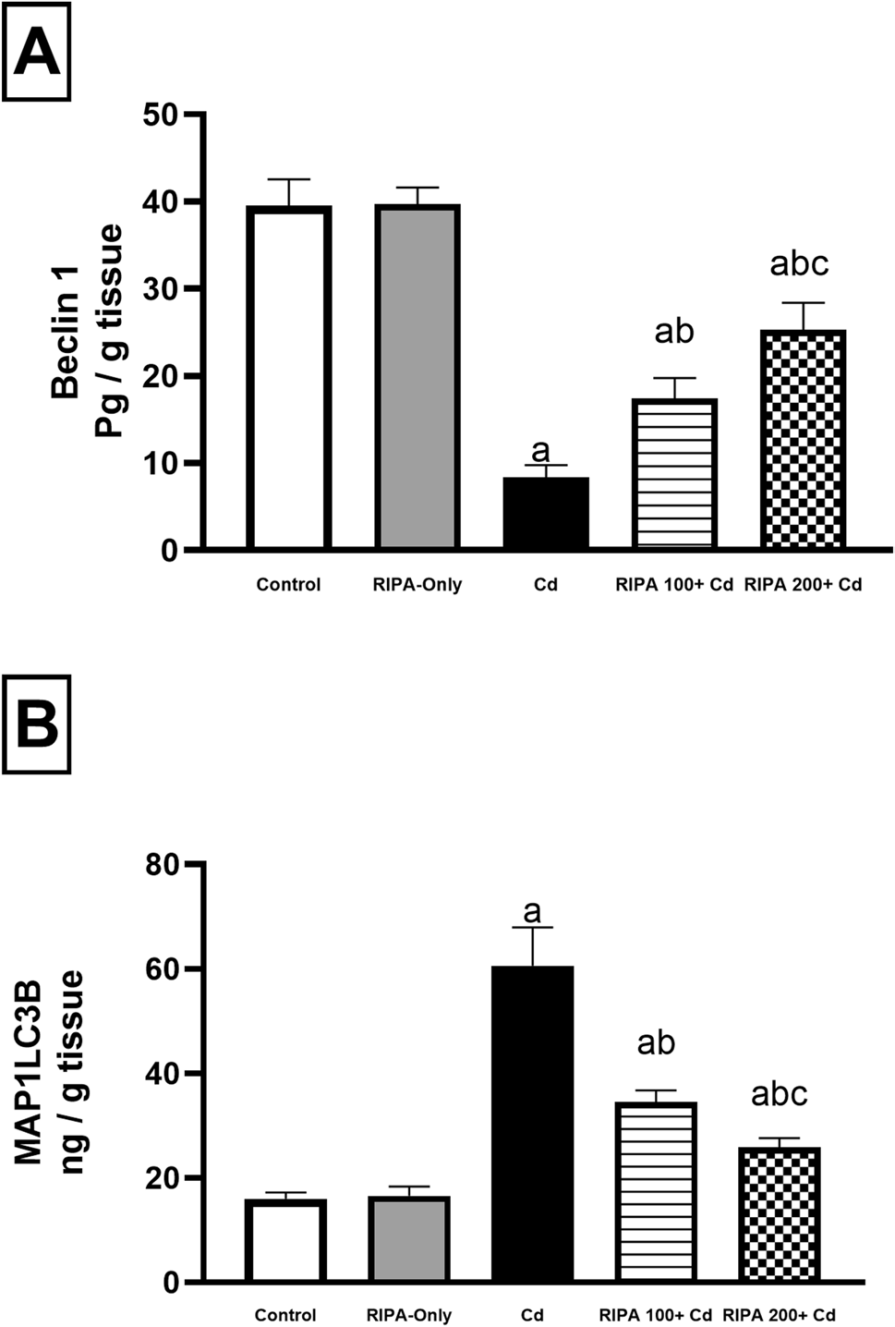
(**A**-**B**) Renoprotective effect of rebamipide (100 and 200 mg/kg/day for 9 days: 7 days before and 2 days after a single intraperitoneal dose of Cadmium chloride [5 mg/kg]) on renal (**A**) Beclin-1 and (**B**) Microtubule-associated proteins 1A/1B light chain 3 (LC3) in cadmium-induced renal toxicity. Values represent the mean ± SD (*n* = 10). Different superscript letters (a, b, c) indicate significant differences among groups (*p* < 0.05, one-way ANOVA followed by Tukey–Kramer post-hoc test)

Administration of cadmium (5 mg/kg) caused a significant reduction in renal Beclin-1 by 78.9% and an increase in microtubule-associated proteins 1A/1B light chain 3 (LC3) by 278.6% compared to the normal control group (*p* < 0.05). Concurrent administration of rebamipide at the low dose (100 mg/kg) increased Beclin-1 by 108.38% and decreased MAP1LC3B by 43% compared to the cadmium-only group. The high dose (200 mg/kg) produced a more pronounced increase in Beclin-1 by 203.2% and decreased MAP1LC3B by 57.9%" Fig. 3. These data suggest that rebamipide modulates cadmium-induced dysregulation of autophagy.

### Effect of rebamipide on renal cadmium concentration

Administration of cadmium (5 mg/kg) caused a significant elevation in renal cadmium concentration, approximately tenfold higher in the cadmium-exposed group compared to controls (*p* < 0.05). Concurrent administration of rebamipide at the low dose (100 mg/kg) decreased cadmium concentration by 46.6% compared to the cadmium-only group. The high dose (200 mg/kg) produced more pronounced reductions in cadmium concentration by 62.1%. Table 2. This indicates that rebamipide may facilitate cadmium excretion or reduce its renal uptake.

**Table 2:** The renoprotective action of rebamipide was evaluated against cadmium-induced renal toxicity. Treatments involved oral administration of two doses: rebamipide at 100 mg/kg/day and 200 mg/kg/day. The dosing regimen spanned nine days in total, commencing one week prior to a single intraperitoneal injection of cadmium (5 mg/kg) and continuing for two days there after. The protective effects were assessed by measuring the Cadmium concentration in the kidney

Parameter	Cd in kidney
Treatment	**Ng/g tissue**
CONT-saline	0.1250 ± 0.01871
Cd (5 mg/kg)	1.490 ± 0.1352^a^
REBA 100 + cd	0.7950 ± 0.06921^ab^
REBA 200 + cd	0.5650 ± 0.06745^abc^

Each value represents the mean and standard deviation of the mean of 10 rats

Different letters (a, b, c) denote statistically significant differences (*p* < 0.05) among the treatment groups as determined by one-way ANOVA followed by the Tukey–Kramer test

### Effect of rebamipide on cadmium nephrotoxicity in rats on the renal histopathology

**Fig. 4:**
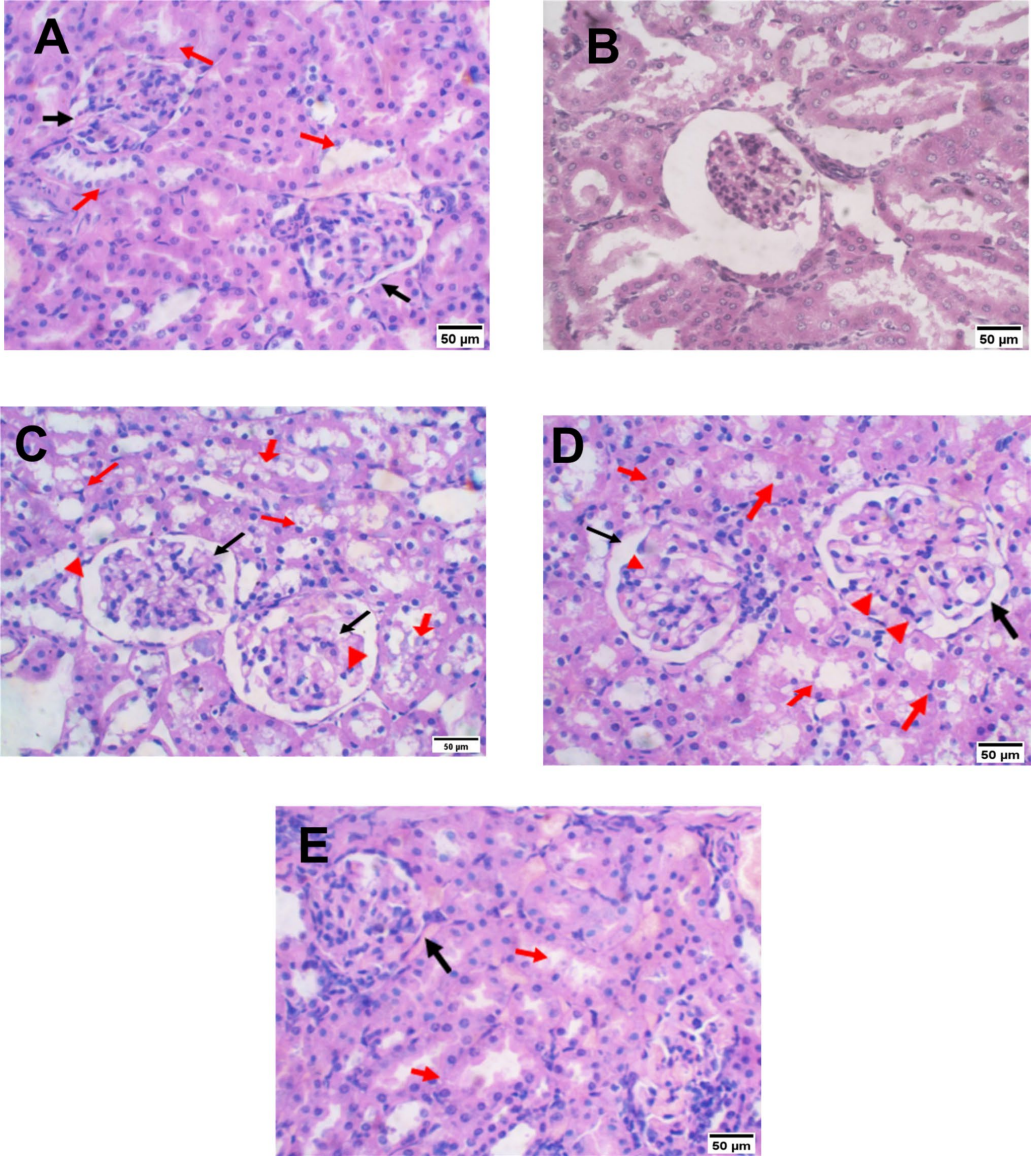
(**A**-**E**) Renoprotective effect of rebamipide (100 and 200 mg/kg/day for 9 days: 7 days before and 2 days after a single intraperitoneal dose of Cadmium chloride [5 mg/kg]) on renal histopathology in cadmium-induced renal toxicity in rats (Hematoxylin & Eosin staining; magnification × 40). **A** Normal control group: showing normal histological structure of glomeruli and tubules. **B** Rebamipide-only group: showing no histopathological alterations, with preserved normal histological structure of glomeruli and tubules. **C** Cadmium group: showing tubular necrosis, loss of brush border, apical vacuolization, and evidence of interstitial inflammation. **D** Rebamipide (100 mg/kg) + Cd group: showing a significant reduction in the extent and severity of interstitial inflammation. **E** Rebamipide (200 mg/kg) + Cd group: kidney tissue restored to normal appearance and arrangement with no evidence of interstitial inflammation, though few tubules show manifestations of mild tubular injury

Histopathological examination of normal kidney tissues revealed the common structure of the kidney Fig. 4A. Administration of rebamipide alone also showed no histopathological alterations Fig. 4B). Administration of cadmium caused flattening of tubular epithelial cells, cellular necrosis, loss of brush border, and vacuolization in the renal cortex. Glomeruli showed thrombi in some capillary lumens, along with evidence of interstitial inflammation (Fig. 4C). The protective effect of rebamipide successfully prevented cadmium-induced damage, and kidney tissues appeared similar to normal. Although concurrent administration of rebamipide at the low dose (100 mg/kg) provided significant protection, some tubules still showed manifestations of moderate tubular injury, indicating partial but not complete protection at this dosage (Fig. 4D). The high dose (200 mg/kg) produced more pronounced reductions in histopathological damage. Most kidney tissue restored its natural architecture, as evidenced by glomeruli showing no signs of injury (Fig. 4E and Table 3).

**Table 3:** The renoprotective action of rebamipide was evaluated against cadmium-induced renal toxicity. Treatments involved oral administration of two doses: rebamipide at 100 mg/kg/day and 200 mg/kg/day. The dosing regimen spanned nine days in total, commencing one week prior to a single intraperitoneal injection of cadmium (5 mg/kg) and continuing for two days thereafter. The protective effects were assessed by measuring the histopathological changes

	N-Normal	Repa only	C-Cadmium	Group 1	Group 2
Kidney	No pathology	No pathology	The kidney showed tubular injury of grade three class III indicates glomerular injury and moderate interstitial inflammation in several foci	The kidney showed Tubular injury with grade three class III indicates glomerular injury and mild interstitial inflammation in one focus	The kidney showed Tubular injury with grade one class zero indicates no glomerular injury or interstitial inflammation

Grade zero: indicated no tubular injury

Grade one: indicated: 25% tubular injury

Grade two: indicated: 50% tubular injury

Grade three: indicated: from 50 to 75% tubular injury

Glomerular scoring:

Class zero: indicated no glomerular injury

Class one: indicated mild glomerular injury

Class two: diffuse mesangial expansion

IIa: mild glomerular injury more than 25%

IIb: severe glomerular injury more than 25%

Class three: glomerular thrombi

## Discussion

It is necessary to monitor environmental pollutants that cause damage to the kidneys [28]. There is no doubt that cadmium (Cd), is highly associated with kidney toxicity [29]. The pathophysiology of renal injury following cadmium administration is complex, and numerous mediators are involved [30]. Cadmium is stored mainly in the proximal tubules of the kidney and can remain in the body for up to 45 years [31, 32].

In our study, a single dose of cadmium (5 mg/kg) caused a significant elevation in serum urea and creatinine levels due to glomerular and tubular kidney injury, a result that agrees with the findings of [33, 34]. The decline in glomerular filtration rate (GFR) is a critical problem in Cd-induced nephropathy[35]. Kidney injury molecule-1 (KIM-1) is an indicator of early kidney injury, and cadmium accumulation in the kidney results in its elevation [36, 37].

The pathophysiology of Cd-induced renal injury is multifaceted, primarily driven by oxidative stress. Cadmium (Cd) accumulation in the kidney causes a decrease in antioxidant enzymes, including glutathione (GSH), catalase (CAT), and superoxide dismutase (SOD), along with increased ROS levels, a result that agrees with the findings of Fang, Xie et al. [38]. Cadmium-induced acute kidney injury is primarily mediated by oxidative stress, which damages cell components [39]. Cadmium can affect thiol-containing proteins within mitochondria, disrupt their activity, and impair mitochondrial metabolic pathways and membrane permeability [40, 41] Metallothionein plays a role in protection from cadmium toxicity, and its synthesis requires cysteine, which may be derived from the breakdown of glutathione [42, 43]. Lipid peroxidation in rats treated with cadmium is initiated and propagated by the increased production of reactive oxygen species (ROS), while the reduction in SOD and CAT activity exacerbates this process by impairing the cellular antioxidant defense [44]. Cadmium upsets the antioxidant defense system of SOD of cells through a number of mechanisms, one of which is the displacement of metals like zinc from their natural binding sites [45]. The increase in MDA is due to enhanced lipid peroxidation caused by free radicals [46]. Lipid peroxidation results in amino acid oxidation, K + ion leakage, the disintegration of biological membranes’ structural and functional integrity, and ultimately cell death [47].

Cadmium-induced nephrotoxicity upregulates the expression of tumor necrosis factor-alpha (TNF-α) and nuclear factor kappa B (NF-κB), a result that agrees with the findings of [48, 49]. The increase in TNF-α and NF-κB is a natural response to elevated reactive oxygen species (ROS) levels after cadmium exposure[50]. Cd-induced kidney damage can be demonstrated by measuring NOS isoenzymes, including inducible nitric oxide synthase (iNOS) and NO itself [51]. The subsequent induction of pro-inflammatory mediators, including iNOS and cyclooxygenase-2 (COX-2), propagates tissue damage [52]. The nephrotoxic effect of NO is mediated either directly by NO itself or indirectly through glutathione depletion, exacerbating glomerular and tubular injury [51, 53, 54]. This interplay between oxidative stress and inflammation creates a vicious cycle of renal damage.

The basic molecular processes of Cd nephrotoxicity appear to be apoptosis and autophagy, according to mounting evidence [55]. Beclin-1 increases the formation of autophagosomes by serving as a platform that connects autophagy-related proteins (ATGs) with the class III phosphatidylinositol 3-kinase (PI3K) complex [56]. Cadmium renal toxicity causes a decrease in autophagy by reducing Beclin-1 activity; these results are in agreement with [27]. The primary protein for identifying autophagy is Microtubule-associated proteins 1A/1B light chain 3 (LC3), which also plays a crucial role in vesicle elongation when double-membrane autophagosomes are formed [57]. Cd exposure increased MAP1LC3 mRNA concentration, which agrees with the findings of [58].

Renoprotective effect of Rebamipide is supported by its antioxidant, anti-apoptotic and anti-inflammatory actions. Rebamipide significantly restored the balance of mitochondrial ROS and anti-oxidant enzymes, both of which are crucial in mitigating the effects of cadmium-induced renal damage, in a dose-dependent manner. Rebamipide increased the activities of the antioxidant enzymes catalase and superoxide dismutase. This led to a reduction in lipid peroxide formation and preserved glutathione (GSH) levels, thereby preventing the increase in superoxide anions and hydrogen peroxide [59]. Superoxide dismutase scavenges the superoxide anion while catalase eliminates hydrogen peroxide [60]. Rebamipide decreased cadmium-induced nephrotoxicity by reducing malondialdehyde (MDA) production and thereby directly protecting cellular membranes from oxidative damage [19, 20]. Rebamipide prevented the disturbance in Cu, Zn-superoxide dismutase and glutathione S-transferase activities [25]. This action is crucial in mitigating the initial insult that triggers downstream pathological events. The nephroprotective mechanisms of Rebamipide depend on its anti-inflammatory activity, which is mediated through the inhibition of NF-κB pathway [61]. This led to a significant reduction in the levels of its downstream effectors, including TNF-α and iNOS [62, 63]. The reduction in NF-κB signaling pathway activity is dose-dependent with rebamipide [23]. These actions highlight its efficacy in disrupting the pro-inflammatory signaling cascade at a pivotal point. A key insight from our work is the ability of rebamipide to restore autophagic homeostasis. Treatment normalized the expression of Beclin-1 and reduced Microtubule-associated proteins 1A/1B light chain 3 (LC3), suggesting a promotion of functional autophagy, which may facilitate the clearance of Cd-damaged cellular components and promote tubular cell survival[64]. This effect on autophagy, coupled with its anti-apoptotic properties, underscores its role in preserving cellular integrity [65]

Rebamipide could reduce the level of serum creatinine and urea and lower KIM-1 expression, a result that agrees with the findings of [66, 67]. The convergence of these mechanisms—antioxidant, anti-inflammatory, and pro-autophagic—translates into significant functional and histological preservation. Treatment with Rebamipide mitigated cadmium-induced nephrotoxicity, resulting in a significant preservation of kidney morphology, an effect consistent with previously reported results. Rebamipide succeeded in overcoming the deterioration of glomerular capillaries, increasing the capsular space, and reducing tubular injury.

## Conclusion

This study demonstrates that rebamipide provides significant protection against cadmium-induced nephrotoxicity. Rebamipide mitigates cadmium-induced kidney toxicity through its antioxidant activity by activating superoxide dismutase and increasing reduced glutathione levels, coupled with the marked reduction of critical inflammatory mediators: kidney injury molecule-1 (KIM-1), tumor necrosis factor-α (TNF-α), nuclear factor kappa B (NF-κB), and inducible nitric oxide synthase (iNOS) in renal tissue. These findings position rebamipide as a promising therapeutic candidate for mitigating chemical-induced kidney injury.

## Data Availability

All data will be available upon request.
